# Herpes simplex virus infects most cell types in vitro: clues to its success

**DOI:** 10.1186/1743-422X-8-481

**Published:** 2011-10-26

**Authors:** Ghadah A Karasneh, Deepak Shukla

**Affiliations:** 1 Department of Ophthalmology and Visual Sciences, University of Illinois at Chicago, College of Medicine, (1855 W. Taylor), Chicago, IL (60612), USA; 2 Department of Microbiology and Immunology, University of Illinois at Chicago, College of Medicine, Chicago, (835 S. Wolcott) IL (60612), USA

**Keywords:** Herpes simplex virus (HSV) type-1 and type-2, HSV entry, Receptors

## Abstract

Herpes simplex virus (HSV) type-1 and type-2 have evolved numerous strategies to infect a wide range of hosts and cell types. The result is a very successful prevalence of the virus in the human population infecting 40-80% of people worldwide. HSV entry into host cell is a multistep process that involves the interaction of the viral glycoproteins with various cell surface receptors. Based on the cell type, HSV enter into host cell using different modes of entry. The combination of various receptors and entry modes has resulted in a virus that is capable of infecting virtually all cell types. Identifying the common rate limiting steps of the infection may help the development of antiviral agents that are capable of preventing the virus entry into host cell. In this review we describe the major features of HSV entry that have contributed to the wide susceptibility of cells to HSV infection.

## Introduction

Herpes simplex viruses (HSV) are part of the alphaherpesvirus subfamily of herpesviruses. There are two types of HSV: type-1 (HSV-1) and type-2 (HSV-2). These viruses are neurotropic capable of infecting the nervous system and causing neurological diseases. Moreover, HSV results in a lifelong infection by establishing latency in the host sensory neurons and replicating in epithelial cells during primary infection and reactivation [1]. The virus is spread and transmitted among humans through physical contact and commonly causes localized mucocutaneous lesions [2]. Oral and ocular lesions are primarily caused by HSV-1 and genital lesions by HSV-2. However, HSV-2 is capable of causing ocular lesions in newborns of HSV-2 infected mothers. In that case, HSV-2 is transmitted to newborns primarily during peripartum period as a result of disrupted membranes, or by direct contact with the mother's vaginal secretions and infected cervix [3,4]. These viruses are also capable of causing more serious diseases, such as blindness, meningitis, and encephalitis [5]. HSV-1 is a leading cause of viral corneal blindness and viral encephalitis in developed countries [6,7].

Unlike many herpesviruses, HSV has low species specificity and a wide host range. It has the unparalleled ability to infect human and nonhuman cells alike [8]. The reason behind this successful story of infection is an accumulation of multiple supporting factors. These include:

• Involvement of several multifunctional HSV glycoproteins in entry.

• Existence of multiple alternative receptors. An array of HSV entry receptors for HSV glycoproteins already exists, and evidence suggests even more unidentified HSV receptors.

• Multiple entry modes. HSV has the ability to enter into host cells by direct fusion with the plasma membrane, or via endocytic pathways. The latter can be pH dependent or independent.

• Multiple spread strategies of HSVs, including: transmission of free virions, movement of HSV along filopodia-like cellular membrane protrusions (surfing) towards the cell body, and lateral cell-to-cell spread.

This review discusses recent advances in the field of HSV entry and highlights the strategies exploited by the virus to infect a wide range of hosts.

### HSV structure

The mature infectious HSV consists of four components from the core outward: an opaque dense core that contains linear double stranded DNA (approximately 152 kB), encoding at least 74 genes [9]. HSV genome is encapsulated within an icosahedral capsid that consists of 162 capsomeres with six different viral proteins (VPs) present on the surface [10]. The capsid is surrounded by a protein layer called the tegument that contains 22 VPs. Finally, an outer envelope that contains 16 membrane proteins, including 12 different proteins that contain oligosaccharide chains (glycoproteins). These glycoproteins are of particular importance for the purpose of this review since their interactions with the host cell surface proteins mediate HSV entry into the cell. These glycoproteins are: gB, gC, gD, gE, gG, gH, gI, gJ, gK, gL, gM, and gN [10-12].

Some of these glycoproteins have been found to exist as heterodimers including the heterodimers gH-gL and gE-gI. Many associate with each other, and have the potential to function as oligomeric complexes [13]. In addition, these glycoproteins are suggested to have distinct size, morphology and distribution in the viral envelope, based on studies that have used the electron microscope, and monoclonal antibodies against the viral glycoproteins gB, gC, and gD. Accordingly, gB forms the most prominent spikes that are about 14 nm long with a flattened T-shaped top, invariably clustered in protrusions of the viral envelope. While gC were up to 24 nm long distributed randomly, and widely spaced. gD seemed to be 8-10 nm long, clustering in a distinct irregular pattern [14].

### HSV entry

**Figure 1:**
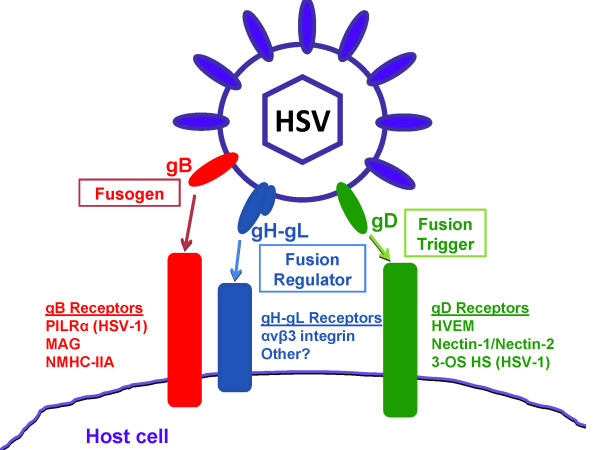
**HSV-1 glycoproteins required for viral entry and their identified receptors**. HSV-1 entry requires the glycoproteins gB, gD, and the heterodimer gH-gL. Some of the receptors are exclusive to HSV-1 including PILRα and 3-OS HS.

HSV entry into host cell is a multistep process that is a result of fusion between the viral envelope and a host cell membrane. It is mediated and modulated by the action of seven HSV glycoproteins along with their interactions with their cognate receptors. These glycoproteins are gB, gC, gD, gH, gK, gL, and gM [1]. However, only four of these glycoproteins (gB, gD, gH, and gL) are necessary and sufficient to allow virus fusion with the plasma membrane of the host cell (Figure 1) [15-18].

The first step in HSV entry is the attachment of HSV through the envelope glycoproteins gB and/or gC to heparan sulfate proteoglycans (HSPG) on the surface of the host cell [19]. The purpose of this interaction is thought to tether the virus to cells in order to concentrate the virus at the cell surface [5]. Although gC enhances HSV binding through its interaction with heparan sulfate (HS), it is not essential for entry [8]. The next step in entry is specific interaction between HSV gD and a gD receptor [20]. Several gD receptors have been identified, and they are discussed in more detail below. This interaction allows for tight anchoring of the virion particle to the plasma membrane of the host cell, and brings both the viral envelope and the cell plasma membrane into close juxtaposition [12]. It is thought that the interaction of gD with one of its receptors triggers a cascade of events that lead to membrane fusion. Structural studies of gD prior to receptor binding and in complex with a receptor suggest that gD undergoes conformational change upon receptor binding, which may transmit an activation signal to gB, and gH/gL leading to membrane fusion. Thus, fusion requires the formation of a multiprotein complex (a fusogenic complex) comprised of gD, gB, and gH/gL [21-25]. A proline-rich region (PRR) of gD has been shown to be important for this process [26]. Whether this region becomes exposed to contact gB and gH/gL upon receptor binding, or this region functions as a flexible joint to expose an unidentified region is still unknown.

gD crystal structure reveals that its ectodomain consists of a V-like immunoglobulin (IgV) core that is wrapped by two topologically and structurally distinct extensions: N-terminus which has the receptors binding sites, and the C-terminus that has a domain required for triggering viral membrane fusion [21,27,28]. Various gD receptors bind distinct binding sites on the N-terminus [29-31]. Soluble gD has been shown to be sufficient to allow the entry of gD-null virus into the host cell [32]. In addition, soluble forms of gD receptors have been also shown to be sufficient to allow wt virus entry into cells lacking gD receptors [33]. These observations suggest that gD binding to its receptor is important to modify gD so that it can trigger fusion. A number of studies support the idea that the C-terminus of gD binds to the N-terminus resulting in an autoinhibitory closed conformation. gD receptor binding results in conformational change where the C-terminus is displaced adopting an open conformation, and thus activating the fusion machinery [26,34].

Although gB does not promote membrane fusion by itself, its crystal structure reveals that gB share some properties with other class I and class II fusion proteins. gB belongs to a newly defined class of fusogens: class III. It is a multidomain trimer that is suggested to undergo a complex and ordered refolding process to drive fusion [35]. Currently solved gB structure is predicted to represent the post-fusion conformation of the protein [36]. It possesses five domains and two linker regions in each protomer of the trimeric ectodomain: (Domain I) has the fusion loop, (domain II) is located in the middle, (domain III) is an α-helical coild coil that represents the core of the protein, (domain IV) is the crown domain that has the epitopes for HSV specific neutralizing antibodies, and (domain V) is the arm domain that consists of a long extension spanning the full length of the protomer and contact to the other two protomers [5,36-38]. The long linker regions are suggested to allow gB conformational change during fusion.

Some investigators suggest gH/gL to have a fusogenic activity by initiating hemifusion [22]. However, a recently resolved crystal structure of HSV-2 gH/gL revealed that gH/gL structure does not resemble any known viral fusogen [39]. Moreover, a recent study, using cell fusion assay demonstrated that gD, a gD recptor and gH/gL heterodimer are unable to induce hemifusion formation [40]. Consistent with these results, a model was proposed where conformational changes in gD, upon its receptor binding, enable it to activate the heterodimer gH/gL into a form that binds to and activates gB fusogenic activity [41]. Thus gH/gL is suggested to act as a regulator of the fusion process by gB rather than a fusogenic glycoprotein [41]. The interaction of the heterodimer gH/gL with gB does not seem to require the presence of these glycoproteins on the same membrane; since cell-cell fusion has been reported when gB and gH-gL were expressed in trans on different cells [41].

### HSPG as an attachment coreceptor

HSV attachment to HS is the first step of HSV infection. HS is abundantly expressed on the surface of almost all cell types as HSPG. Additionally, the highly sulfated HS possesses negative charges making it suitable to interact with the positively charged viral glycoproteins [42]. Evidence for this interaction stems from the observations that HSV attachment to cell lines that are defective in HS biosynthesis, but not chondroitin sulfate (CS) biosynthesis is reduced by 85% causing a significant reduction in infectivity in these cell lines [43]. Moreover, soluble heparin, which is closely related to HS, binds HSV, causing an inhibitory effect on HSV binding to host cells [44]. Enzymatic digestion of HS reduces HSV infection [45].

The viral glycoproteins gB and gC are involved in the attachment to HS. The affinity of gB and gC to HSPG is different for HSV-1 and HSV-2. While HSV-1 gC has critical role in HSPG attachment during HSV-1 entry, HSV-2 gB is the key glycoprotein for HSV-2 attachment to HSPG [46,47]. The difference in HSV-1 and HSV-2 gB and gC affinity to HS is suggested to influence several biological activities including sensitivity to polyanionic and polycationic substances [48]. Although viral attachment to HS enhances the infection, the lack of gC on the viral envelope, or the lack of HS on cells lowers the efficiency of the infection, but does not prevent it [49,50]. The lack of gB prevents the infection, primarily because of its critical role during membrane fusion of the virus. In addition to differences between HSV-1 and HSV-2 in the key glycoprotein that interacts with HS during virus attachment, it has been shown that HSV-1 and HSV-2 interact differently with the various structural features of cell surface HS [51]. HS binding can also play a role in the virus's ability to form larger plaques since a mutant virus deleted for a putative HS binding lysine-rich sequence in gB (residues 68-76) showed reduced plaque sizes [52].

Although the role of HS as an attachment receptor has been intensively studied, little is known about the contribution of the core protein that carries the HS chains. It is known that several families of HSPG have been identified, and one major family is the syndecan family of HSPG [53]. Recently, work from our lab has shown that two members of the syndecan family of HSPG (syndecan-1 and syndecan-2) play a role during HSV entry [54]. The downregulation of these syndecans using specific small interfering RNA (siRNA) resulted in a significant reduction in HSV entry and plaque formation. These results were confirmed using antibodies blocking assay, where antibodies against syndecans were capable of inhibiting viral entry [54]. Interestingly, HSV infection resulted in the upregulation of syndecan-1 and syndecan-2 expression on the cell surface and at the protein level [54]. This observation strongly suggests that these HSPG are involved in the infection, and most probably beyond the attachment step of the infection. Future studies will determine the exact contribution of the various members of the syndecan family in HSV infection. Table 1 lists HSV know entry receptors for some of the tested human cell lines.

**Table 1:** HSV known entry receptors for some of the tested human cell lines

Cell line	Major entry receptor	Other expressed receptors	References
Primary human trabecular meshwork (HSV-1)	HVEM	3-OST-3 (probably 3-OS HS)	[62]
**Primary human corneal fibroblasts (HSV-1)**	**3-OS HS**	**HVEM**	[73]
Primary human corneal fibroblasts (HSV-2)	HVEM		[123]
**Human conjunctival epithelium (HSV-1)**	**Nectin-1, HVEM**	**3-OS HS**	[124]
Retinal pigment epithelium (HSV-1)	Nectin-1	HVEM, 3-OS HS	[96]
**Human corneal epithelium (HSV-1)**	**Nectin-1**	**HVEM, PILRα**	[125]
Retinal pigment epithelium (HSV-2)	Nectin-1	HVEM, PILRα	[126]
**Radial glial cells and Cajal-Retzius cells**	**Nectin-1**		[127]
Soma and processes of central and peripheral neurons, ependymal cells, choroid plexus epithelium, vascular endothelium, meningothelial cells (HSV-1, HSV-2)	Nectin-1		[128]
**Human Mesenchymal Stem Cells (HSV-1)**	**3-OS HS**	**Nectin-1, HVEM**	[129]

### gD receptors

Several lines of evidence suggested that gD is capable of interacting with a cellular receptor. Firstly, the recognition of a phenomenon called gD-mediated restriction to infection or interference. Cells that constitutively express gD become resistant to infection [55,56]. Secondly, truncated soluble form of gD binds to cells until reaching a saturation level [57]. Through generating viral mutants that were able to infect gD expressing cells, the presence of multiple gD receptors was predicted [58]. Currently, there are three classes of gD receptors that belong to structurally unrelated molecular families.

#### Herpesvirus entry mediator (HVEM)

Also called herpesvirus entry mediator A (HveA), is the first gD receptor identified through screening HSV resistant cells transfected with human cDNA libraries [59]. HVEM is a member of the tumor necrosis factor (TNF) receptor family and a regulator of immune responses as part of its normal physiological functions [60]. It binds to HSV gD and mediates HSV entry into entry resistant Chinese hamster ovary (CHO) cells [59,61]. HVEM is expressed in a variety of cell types including T and B lymphocytes, other leucocytes, epithelial cells, fibroblasts and trabecular meshwork and human tissues including the lung, liver, kidney and to less extent in the brain [19,59,62].

#### Nectin-1 and nectin-2

They were first described in literature as poliovirus receptor-related protein 1 and 2 (Prr1 and Prr2) and later renamed as HveC and HveB and eventually nectin-1 and nectin-2 after the discovery of their roles in cell adhesion [63-67]. These cell surface proteins belong to the four membered nectin family of the immunoglobulin superfamily and only nectin-1 and nectin-2 from this family have been shown to mediate HSV entry through their interaction with gD [61,65,66,68].

Nectin-1 has been shown to serve as a receptor for all tested HSV-1 and HSV-2 strains. It is broadly expressed in a wide range of human tissues (e.g. central nervous system (CNS), ganglia, skin, trachea, prostate, thyroid, and liver) and cell lines (e.g. epithelial, endothelial, fibroblastic, keratinocytes, haematopoietic and neuroblastoma) [12]. However, nectin-2, that shares 30% homology with nectin-1 at the protein level, serves as a receptor only for HSV-2 and some unrestricted HSV-1 mutants that do not exhibit gD-mediated restriction of infection. Nectin-2 is considered a low efficiency receptor; therefore, cells expressing nectin-2 requires high multiplicity of infection in order to be infected. The reason of that is thought to be a result of weak physical interaction between nectin-2 and gD (HSV-2 gD, or HSV-1 gD mutants) [66,68]. Nectin-2 is expressed in numerous human tissues (e.g. placenta, kidney, lung, prostate, pancreas, and thyroid) and human cell lines (e.g. epithelial, endothelial, and neuronal) [12].

#### 3-O-Sulfated heparan sulfate proteoglycan (3-OS HS)

3-OS HS is a highly sulfated form of HS that has been shown to serve as a HSV-1 gD receptor, but fails to bind to HSV-2 gD [69]. The 3-O-sulfotransferases family of enzymes is responsible for the generation of the 3-O-sulfation, where each isoform of these enzyme is capable of generating its unique 3-OS HS. 3-OS HS generated by all the isoforms of the 3-O-sulfotransferases, except for one, are capable of binding gD and mediating virus entry. These enzymes have a distinct expression pattern in cells and tissue making them regulators of HS functions [70].

Using soluble 3-OS HS, it has been shown that 3-OS HS is capable of triggering not only virus entry, but also HSV-1 induced cell-cell fusion [71]. Furthermore, the downregulation of a prerequisite for the formation of 3-OS HS; 2-O-sulfation, was found to significantly inhibit HSV-1 binding, entry and virus induced cell-cell fusion [72]. 3-OS HS is expressed in less variety of tissues and cell lines compared to nectin-1. It is found to be expressed in these tissues: liver, placenta, heart, kidney, and pancreas. It is also expressed by the human endothelial cells [12]. In addition, 3-OS HS appears to play a major role in HSV-1 entry into primary cultures of corneal fibroblast [73].

### gB receptors

gB is known for its role in attaching to HSPG for tethering the virus to the cell surface, as well as its crucial role during membrane fusion of the virus. Although virus attachment to HS via gB and gC enhances the infection, the virus can still infect cells deficient in HS synthesis [74]. Using soluble gB, it has been shown that gB is capable of binding to HSPG deficient cells, and block virus entry, suggesting the presence of a gB receptor [75]. Recently three gB receptors have been identified where gB is capable of interacting with these receptors mediating HSV-1 infection.

#### Paired immunoglobulin-like type 2 receptor-α (PILRα)

PILR is one of the paired receptor families. It is expressed mainly in immune cells where one receptor in the family has activating function, while another receptor in the family mediates inhibitory functions. While inhibitory receptors generally recognize self-antigens such as MHC molecules, activating receptors do not recognize self-antigens. Pathogens may utilize the inhibitory receptors to evade the immune system. PILRα has an immunoreceptor tyrosine-based inhibition motif (ITIM) that delivers inhibitory effects. Expression of PILRα in HSV resistant CHO cells renders these cells susceptible to the virus. Moreover, treating susceptible cells with anti-PILRα or anti-HVEM blocked HSV-1 infection, indicating that both gB receptor and gD receptor are required for HSV infection [76]. Interestingly, PILRα has been shown to confer susceptibility to HSV-1 entry, as well as other alphaherpesviruses, including pseudorabies virus, but not HSV-2 into HSV resistant CHO cells [77]. The association of PILRα with HSV-1 gB depends on the presence of sialylated O-glycans on gB with two threonine residues on gB found to be essential for principal O-glycans addition to gB [78].

#### Myelin-associated glycoprotein (MAG)

MAG (also called Sialic-acid-binding Ig-like lectin (Siglec)) is another paired receptor that has 5-12% homology with PILRα [79]. It is localized in the periaxonal space in glial cells suggesting its importance in regulating myelin-axon interactions, including myelination, initiation, and myelin integrity maintenance. Using MAG-/- mice, MAG has been shown to function as an inhibitor of axonal regeneration [80]. MAG associates with HSV-1 gB as well as varicella-zoster virus (VZV) gB and confers susceptibility to HSV-1 and VZV in MAG-transfected promyelocytes and oligodendroglial cells respectively [79]. Since MAG is not naturally expressed in epithelial cells and neuronal cells which are considered prime targets for HSV-1 and VZV, MAG is not thought to serve as a major receptor for these viruses. However, both HSV-1 and VZV infect glial cells in the acute phase of infection, suggesting that MAG might be involved in the neurological disorders caused by HSV-1 and VZV.

#### Non-muscle myosin heavy chain IIA (NMHC-IIA)

NM II binds to actin and has actin cross-linking and contractile characteristics. It is a key protein in the control of many events that are involved in cell reshaping and movement; including cell migration, cell adhesion and cell division. NM-II is composed of two heavy chains, two regulatory light chains, and two essential light chains. NM IIA is one isoform of the NM II protein [81]. NMHC-IIA was identified as an HSV-1 gB receptor using a tandem affinity-purification approach with a membrane-impermeable crosslinker coupled with mass-spectrometry-based proteomics technology [82].

NMHC-IIA has been shown to physically interact with HSV-1 gB, and mediate HSV-1 infectivity both *in vitro *and *in vivo*. Human promyelocytic HL60 cells stably expressing high levels of NMHC-IIA exhibited a significantly higher susceptibility to HSV-1 infection compared to cells expressing low levels of NMHC-IIA. NMHC-IIA role as an entry receptor was also exhibited in naturally permissive cells that express NMHC-IIA endogenously. NMHC-IIA functions primarily in the cytoplasm. However, interestingly, NMHC-IIA cell surface expression was shown to be induced after HSV-1 adsorption at 4°C that was followed by a shift in temperature to 37°C [82]. While PILRα and MAG expression is limited to certain types of cells, NMHC-IIA is ubiquitously expressed in numerous human tissues and cell types, suggesting its important role as the functional HSV-1 gB receptor [81,82].

HSV entry has been closely associated with actin-cytoskeleton reorganization [83]. For example, it has been shown that HSV exposure induces the formation of filopodia-like cell membrane protrusions, on which HSV has the ability to bind and move toward the cell body. This movement of HSV on filopodia is termed surfing, and believed to be a spread strategy of the virus. In addition, HSV glycoprotein gB was found to be critical for virus surfing [84]. Since NMHC-IIA binds to actin, and is involved in many events controlling cell movement and reshaping, it is quite possible that virus surfing on filopodia is mediated by gB binding to NMHC-IIA. More studies are needed to investigate the contribution of NMHC-IIA in virus surfing.

#### Lipid-raft associated gB receptor

An HSV gB lipid-raft-associated receptor has also been proposed by the observation that gB, but not gC, gD, or gH associates with glycolipid-enriched membranes (DIG) that represent raft-containing fractions. Since gC does not associate with DIG, gB association with DIG is suggested to be either heparan sulfate independent, or heparan sulfate dependent where gB is associating with specific type of HSPG, or has a differential effect compared to gC association with HSPG [85].

### gH-gL receptors

Many lines of evidence support the presence of gH-gL receptor; nevertheless, the contribution of this gH-gL-receptor interaction to HSV infection is not yet fully understood. An observation was made in our lab where the cellular expression of gH-gL confers resistance to HSV-1 entry, indicating that gH-gL on the cell surface may result in sequestering the available cellular gH-gL receptor, perturbing the entry process of the virus [86]. Soluble gH-gL has been shown to bind to αvβ3 integrin through a potential integrin-binding motif, Arg-Gly-Asp (RGD), in gH [87]. However, mutating RGD to triple RGE (Arg-Gly-Glu) does not affect HSV-1 entry [88]. gH-gL was also found to bind to cells independently of αvβ3, and that binding was found to be important for HSV entry and membrane fusion [89]. Additional studies are important to identify the presence, and understand the significance of possible gH receptors during HSV infection.

### B5 protein

Using expression cloning, *hfl*-B5 gene was isolated that encodes a cellular protein found to be involved in HSV infection. B5 is a type-2 membrane protein that has an extracellular heptad repeat potentially capable of forming an α-helix for coiled-coils. B5 is ubiquitously expressed on many human cell lines. Transfecting porcine renal epithelial cells that are naturally resistant to HSV rendered these cells susceptible to the virus, which made B5 protein a candidate HSV receptor [90]. It was proposed that B5 α-helix might interact with viral proteins containing α-helices such as gH to facilitate membrane fusion. However, a recent study revealed that B5 role in HSV infection is not during HSV entry, but during HSV proteins translation. B5 silencing did not affect entry markers including intracellular viral capsids delivery and viral tegument protein nuclear transport. On the other hand, B5 silencing was found to inhibit viral immediate early proteins translation [91].

### The design of new antivirals utilizing HSV receptors

Advances in the field of HSV receptors provide new strategies for the generation of anti-HSV agents. Experiments done to identify the major HSV entry receptor in various cell types have exploited assays including antibody blocking assays, and the down regulation of HSV receptors utilizing siRNA [2,62,73]. These assays indicated that HSV infection can be inhibited by blocking the viral entry receptors. Copeland and colleagues have generated a 3-OS HS octasaccharide that has the ability to inhibit HSV-1 entry [92]. Recently, our lab has isolated 12-mer peptides that bind specifically to HS, or 3-OS HS, and block HSV-1 entry. Interestingly, peptides isolated against 3-OS HS exhibited the ability to inhibit the entry of not only HSV-1, but also some divergent members of herpesvirus family including cytomegalovirus (CMV) and human herpesvirus-8 (HHV-8) [93].

### HSV entry modes

Recent studies have shown that HSV can follow different entry routes. Two major entry routes include: (I) a pH independent fusion with the plasma membrane of the host cell (II) endocytosis that may be phagocytosis-like where the virus triggers the fusion with the phagocytic membrane [83,94]. This entry route may not always be pH dependent. Although all HSV glycoproteins function at neutral and low pH, gB undergoes minor conformational changes under low pH, the consequences of these changes are not yet known [95]. Anti-acidification drugs such as bafilomycin A enable the identification of pH dependence during endocytosis.

The differential entry route that HSV can follow is cell type-specific. For example, HeLa, human retinal pigment epithelial cells (RPE), and the CHO cell line expressing nectin-1 gD receptor allow HSV entry through low pH endocytic pathway. However, the monkey kidney epithelial cells (vero) allow HSV entry through the direct fusion with plasma membrane of the host cell [96,97]. Regardless of the entry route followed, HSV enters host cells by inducing fusion between the viral envelope and the host cell membrane.

Very little is known about what causes these differential entry routes. Since it is cell type specific, it is suggested that cellular determinants are responsible for choosing the viral entry route into the host cell. The contribution of gD receptors in determining the entry mode is elusive. One study has shown that co-culturing cells expressing gD with cells expressing nectin-1 resulted in the downregulation of nectin-1 in cells where HSV enters by endocytosis but not in cells where HSV enters at the plasma membrane. This suggested that gD mediated internalization of nectin-1 directs HSV to an endocytic mode of entry to cells [98]. Also, a mutant HSV strain enters CHO-nectin-1 cells via the endocytic pathway, but fuses at the plasma membrane of CHO-nectin-2 cells [99]. On the other hand, in another study, ^35^S-HSV uptake from the surface of CHO-nectin-1 cells was similar to that of CHO cells that lack any known gD receptors, indicating that the presence of nectin-1 does not promote endocytic uptake of HSV into CHO cells [100].

Recently, several other cellular determinants have been suggested to be involved in determining HSV entry route. The gB receptor PILRα is one of these cellular determinant, where it is found to direct HSV to the fusion at the plasma membrane mode of entry. While HSV uptake to CHO cells and CHO-nectin-1 cells is mediated by endocytosis, HSV entry into CHO cells expressing PILRα was found to be mediated by fusion at the cell surface [77]. Suggesting that an alternative entry mode for HSV was produced by the expression of the gB receptor PILRα. The integrin αvβ3 was also found to be involved in directing the viral route of entry. Overexpressing αvβ3 in CHO-nectin-1 cells, that naturally lack αvβ3, modifies the route of entry to an acidic compartment dependent on cholesterol-rich rafts and dynamin2. Moreover, overexpressing αvβ3 in J-nectin-1 and 293T cells modifies the route of HSV entry from neutral compartments to acidic compartments dependent on cholesterol-rich rafts and dynamin2 [101].

### HSV successful infection of various hosts

Although HSV is considered a human herpesvirus, it has a wide species host range, and thus has the ability to infect animals and cell cultures of various species [12]. There are reports of HSV experimentally infecting mice, rabbits, guinea pigs, zebrafish, and cultured Madin-Darby canine kidney (MDCK) cells [102-106]. The ability to experimentally infect non-habitual species by HSV suggests that HSV entry requirements, including the various receptors and entry modes, are quite commonly available and accessible on the cells of various host species.

Another point that might explain HSV broad species host range is that HSV can exploit, as receptors, animal homologues of HSV receptors. For example, the murine homologue of human nectin-1δ isoform has a > 90% identity with its human counterpart, and act as a species non-specific entry receptor of HSV, pseudorabies virus (PrV), and bovine herpesvirus-1 (BHV-1). Interestingly, soluble murine nectin-1δ does not bind HSV gD at a detectable level, although it interacts physically with the virion [107]. On the other hand, the murine homologue of human nectin-1 was found to be capable of mediating PrV entry, but not HSV entry [108]. Other examples are the murine and zebrafish homologs of the 3-O-sulfotransferases enzymes responsible for modifying HS generating the HSV-1 gD receptor 3-OS HS [109,110]. Interestingly, the expression of the zebrafish homolog of 3-O-sulfotransferase-3 isoform into the entry resistant CHO cells and zebrafish fibroblasts has been shown to mediate HSV-1 entry and spread [111].

Various species that are susceptible to HSV provide important animal models for HSV research. The mouse model has been widely used in HSV entry, pathogenesis and anti-viral research, while guinea pigs and rabbits are suitable animal models for HSV latency research [112]. Since zebrafish has a fully developed immune system, it has been suggested that this animal model be utilized to study HSV interactions with the immune system [105]. These various specious have been infected experimentally for research purposes, most of them are not naturally infected with HSV. However, there are some rare cases where some of these animals get the infection naturally. For example, there are two reported cases of rabbits naturally infected with HSV-1 leading to encephalitis [113,114].

An important application of HSV animal models is the development of an effective therapeutic anti-herpetic vaccine capable of inhibiting viral reactivation. Several studies have suggested that a crucial element for the generation of anti-herpetic vaccine, is a cellular response specific to HSV, where Interferon-γ (+) (IFN-γ(+)) CD8^+ ^T cells seem to suppress spontaneous reactivation of the latent virus [115,116]. The mouse model has been extensively utilized to study the various aspects of HSV infection, including virus entry and replication [117,118]. Although HSV establishes latency in mice neural tissues and reactivates upon stimulation, spontaneous sporadic viral reactivation does not occur in mice [119,120]. This is an important point for the development of anit-HSV vaccine, since HSV reactivation may result in serious diseases including the blinding herpetic keratitis. Therefore, it has been suggested that the mouse model is not the suitable model for studying the effectiveness of anti-HSV vaccines that inhibit viral reactivation [121]. Two other animal models have been suggested to study the effectiveness of anti-HSV vaccine that inhibits spontaneous viral reactivation: rabbit and genie pig. HSV is capable of establishing latent infection in these animal models, and reactivate spontaneously causing disease, similar to HSV infection in human [121]. Recently, a Human Leukocyte Antigen (HLA) transgenic rabbit model has been introduced for preclinical evaluation of human CD8(+) T cell epitope-based vaccines against ocular HSV infection [122].

## Conclusions

The ability of HSV to productively infect a wide range of hosts and cell types suggests that HSV has evolved to use multiple receptors and pathways to facilitate entry into multiple cell types. Regardless of HSV entry receptors or pathways utilized, HSV entry into host cell has common features among various routes of virus entry, including HSV fusion with the plasma membrane of the host cell. This indicates that HSV might recognize structural features of receptors that are conserved among various human and animal cell types. The presence of multiple entry receptors and pathways could be the reason of the wide range of hosts and cell types that can be infected by HSV. However this does not answer questions like: what dictates the utilized entry pathway and cellular receptors by HSV? This is particularly important in situations where more than one entry receptor is available for the virus to use on the host cell surface. Moreover, do the various combinations of gB and gD receptors utilized by HSV affect the entry and/or the infection process? Answers to such questions require conducting more studies to fully understand the process of HSV entry into host cell.

Recent advances in the field of HSV cellular receptors and HSV entry glycoproteins' structures, interactions and functions have broadened our understanding of HSV entry into the cell. Such advances will definitely help in the process of developing potent HSV vaccines and anti-HSV drugs. The huge prevalence of HSV in the human population worldwide which increases the risk of acquiring HSV related diseases, including blindness, genital herpes, encephalitis, meningitis, especially in immune compromised patients and infants, urges for such development in the anti-HSV therapies.

## Abbreviations

BHV-1: bovine herpesvirus-1; CHO: Chinese hamster ovary; CMV: Cytomegalovirus; CNS: central nervous system; CS: chondroitin sulfate; DIG: glycolipid-enriched membranes; gB-gN: glycoprotein B-N; HHV-8: Human herpesvirus-8; HLA: Human Leukocyte Antigen; HS: heparan sulfate; HSPG: heparan sulfate proteoglycans; HSV-1 and HSV-2: Herpes simplex virus type-1 and type-2; HveA: herpesvirus entry mediator A; HveB: herpesvirus entry mediator B; HveC: herpesvirus entry mediator C; HVEM: Herpesvirus entry mediator; IFN: Interferon; IgV: V-like immunoglobulin; ITIM: immunoreceptor tyrosine-based inhibition motif; MAG: Myelin-associated glycoprotein; MDCK: Madin-Darby canine kidney; NMHC-IIA: Non-muscle myosin heavy chain IIA; PILRα: paired immunoglobulin-like type 2 receptor-α; PRR: proline-rich region; Prr1 and Prr2: poliovirus receptor-related protein 1 and 2; PrV: pseudorabies virus; RPE: retinal pigment epithelial cells; Siglec: Sialic-acid-binding Ig-like lectin; siRNA: small interfering RNA; TNF: tumor necrosis factor; VPs: viral proteins; VZV: varicella-zoster virus; 3-OS HS: 3-O-Sulfated heparan sulfate proteoglycan; 3-OST-3: 3-O-sulfotransferases isoform 3

## Competing interests

The authors declare that they have no competing interests.

## Authors' contributions

GK and DS contributed to and edited the manuscript. Both authors read and approved the final manuscript.

## References

[B1] Heldwein EE Krummenacher C Entry of herpesviruses into mammalian cells Cell Mol Life Sci 2008 65 11 1653 1668 10.1007/s00018-008-7570-z 18351291 PMC11131758

[B2] Akhtar J Shukla D Viral entry mechanisms: cellular and viral mediators of herpes simplex virus entry FEBS J 2009 276 24 7228 7236 10.1111/j.1742-4658.2009.07402.x 19878306 PMC2801626

[B3] Annunziato PW Gershon A Herpes simplex virus infections Pediatr Rev 1996 17 12 415 423 8973122

[B4] Jacobs RF Neonatal herpes simplex virus infections Semin Perinatol 1998 22 1 64 71 10.1016/S0146-0005(98)80008-6 9523400

[B5] Connolly SA Jackson JO Jardetzky TS Longnecker R Fusing structure and function: a structural view of the herpesvirus entry machinery Nat Rev Microbiol 2011 9 5 369 381 10.1038/nrmicro2548 21478902 PMC3242325

[B6] Herpetic Eye Disease Study Group Acyclovir for the prevention of recurrent herpes simplex virus eye disease N Engl J Med 1998 339 300 306 10.1056/NEJM199807303390503 9696640

[B7] Shoji H Azuma K Nishimura Y Fujimoto H Sugita Y Eizuru Y Acute viral encephalitis: the recent progress Intern Med 2002 41 6 420 428 10.2169/internalmedicine.41.420 12135172

[B8] Spear PG Longnecker R Herpesvirus entry: an update J Virol 2003 77 19 10179 10185 10.1128/JVI.77.19.10179-10185.2003 12970403 PMC228481

[B9] McGeoch DJ Rixon FJ Davison AJ Topics in herpesvirus genomics and evolution Virus Res 2006 117 1 90 104 10.1016/j.virusres.2006.01.002 16490275

[B10] Diefenbach RJ Miranda-Saksena M Douglas MW Cunningham AL Transport and egress of herpes simplex virus in neurons Rev Med Virol 2008 18 1 35 51 10.1002/rmv.560 17992661

[B11] Mettenleiter TC Budding events in herpesvirus morphogenesis Virus Res 2004 106 2 167 180 10.1016/j.virusres.2004.08.013 15567495

[B12] Campadelli-Fiume G Cocchi F Menotti L Lopez M The novel receptors that mediate the entry of herpes simplex viruses and animal alphaherpesviruses into cells Rev Med Virol 2000 10 5 305 319 10.1002/1099-1654(200009/10)10:5<305::AID-RMV286>3.0.CO;2-T 11015742

[B13] Handler CG Eisenberg RJ Cohen GH Oligomeric structure of glycoproteins in herpes simplex virus type 1 J Virol 1996 70 9 6067 6070 8709230 10.1128/jvi.70.9.6067-6070.1996 PMC190628

[B14] Stannard LM Fuller AO Spear PG Herpes simplex virus glycoproteins associated with different morphological entities projecting from the virion envelope J Gen Virol 1987 68 Pt 3 715 725 3029300 10.1099/0022-1317-68-3-715

[B15] Turner A Bruun B Minson T Browne H Glycoproteins gB, gD, and gHgL of herpes simplex virus type 1 are necessary and sufficient to mediate membrane fusion in a Cos cell transfection system J Virol 1998 72 1 873 875 9420303 10.1128/jvi.72.1.873-875.1998 PMC109452

[B16] Pertel PE Fridberg A Parish ML Spear PG Cell fusion induced by herpes simplex virus glycoproteins gB, gD, and gH-gL requires a gD receptor but not necessarily heparan sulfate Virology 2001 279 1 313 324 10.1006/viro.2000.0713 11145912

[B17] Muggeridge MI Characterization of cell-cell fusion mediated by herpes simplex virus 2 glycoproteins gB, gD, gH and gL in transfected cells J Gen Virol 2000 81 Pt 8 2017 2027 10900041 10.1099/0022-1317-81-8-2017

[B18] Campadelli-Fiume G Amasio M Avitabile E Cerretani A Forghieri C Gianni T Menotti L The multipartite system that mediates entry of herpes simplex virus into the cell Rev Med Virol 2007 17 5 313 326 10.1002/rmv.546 17573668

[B19] Spear PG Herpes simplex virus: receptors and ligands for cell entry Cell Microbiol 2004 6 5 401 410 10.1111/j.1462-5822.2004.00389.x 15056211

[B20] Shukla D Spear PG Herpesviruses and heparan sulfate: an intimate relationship in aid of viral entry J Clin Invest 2001 108 4 503 510 11518721 10.1172/JCI13799 PMC209412

[B21] Carfí A Willis SH Whitbeck JC Krummenacher C Cohen GH Eisenberg RJ Wiley DC Herpes simplex virus glycoprotein D bound to the human receptor HveA Mol Cell 2001 8 1 169 179 10.1016/S1097-2765(01)00298-2 11511370

[B22] Subramanian RP Geraghty RJ Herpes simplex virus type 1 mediates fusion through a hemifusion intermediate by sequential activity of glycoproteins D, H, L, and B Proc Natl Acad Sci USA 2007 104 8 2903 2908 10.1073/pnas.0608374104 17299053 PMC1815279

[B23] Gianni T Amasio M Campadelli-Fiume G Herpes simplex virus gD forms distinct complexes with fusion executors gB and gH/gL in part through the C-terminal profusion domain J Biol Chem 2009 284 26 17370 17382 10.1074/jbc.M109.005728 19386594 PMC2719377

[B24] Atanasiu D Whitbeck JC Cairns TM Reilly B Cohen GH Eisenberg RJ Bimolecular complementation reveals that glycoproteins gB and gH/gL of herpes simplex virus interact with each other during cell fusion Proc Natl Acad Sci USA 2007 104 47 18718 18723 10.1073/pnas.0707452104 18003913 PMC2141843

[B25] Avitabile E Forghieri C Campadelli-Fiume G Complexes between herpes simplex virus glycoproteins gD, gB, and gH detected in cells by complementation of split enhanced green fluorescent protein J Virol 2007 81 20 11532 11537 10.1128/JVI.01343-07 17670828 PMC2045520

[B26] Fusco D Forghieri C Campadelli-Fiume G The pro-fusion domain of herpes simplex virus glycoprotein D (gD) interacts with the gD N terminus and is displaced by soluble forms of viral receptors Proc Natl Acad Sci USA 2005 102 26 9323 9328 10.1073/pnas.0503907102 15972328 PMC1166633

[B27] Whitbeck JC Muggeridge MI Rux AH Hou W Krummenacher C Lou H van Geelen A Eisenberg RJ Cohen GH The major neutralizing antigenic site on herpes simplex virus glycoprotein D overlaps a receptor-binding domain J Virol 1999 73 12 9879 9890 10559300 10.1128/jvi.73.12.9879-9890.1999 PMC113037

[B28] Zago A Jogger CR Spear PG Use of herpes simplex virus and pseudorabies virus chimeric glycoprotein D molecules to identify regions critical for membrane fusion Proc Natl Acad Sci USA 2004 101 50 17498 17503 10.1073/pnas.0408186101 15583135 PMC536050

[B29] Whitbeck JC Connolly SA Willis SH Hou W Krummenacher C Ponce de Leon M Lou H Baribaud I Eisenberg RJ Cohen GH Localization of the gD-binding region of the human herpes simplex virus receptor, HveA J Virol 2001 75 1 171 180 10.1128/JVI.75.1.171-180.2001 11119586 PMC113910

[B30] Yoon M Zago A Shukla D Spear PG Mutations in the N termini of herpes simplex virus type 1 and 2 gDs alter functional interactions with the entry/fusion receptors HVEM, nectin-2, and 3-O-sulfated heparan sulfate but not with nectin-1 J Virol 2003 77 17 9221 9231 10.1128/JVI.77.17.9221-9231.2003 12915538 PMC187404

[B31] Manoj S Jogger CR Myscofski D Yoon M Spear PG Mutations in herpes simplex virus glycoprotein D that prevent cell entry via nectins and alter cell tropism Proc Natl Acad Sci USA 2004 101 34 12414 12421 10.1073/pnas.0404211101 15273289 PMC515077

[B32] Cocchi F Fusco D Menotti L Gianni T Eisenberg RJ Cohen GH Campadelli-Fiume G The soluble ectodomain of herpes simplex virus gD contains a membrane-proximal pro-fusion domain and suffices to mediate virus entry Proc Natl Acad Sci USA 2004 101 19 7445 7450 10.1073/pnas.0401883101 15123804 PMC409938

[B33] Kwon H Bai Q Baek HJ Felmet K Burton EA Goins WF Cohen JB Glorioso JC Soluble V domain of Nectin-1/HveC enables entry of herpes simplex virus type 1 (HSV-1) into HSV-resistant cells by binding to viral glycoprotein D J Virol 2006 80 1 138 148 10.1128/JVI.80.1.138-148.2006 16352538 PMC1317534

[B34] Krummenacher C Supekar VM Whitbeck JC Lazear E Connolly SA Eisenberg RJ Cohen GH Wiley DC Carfí A Structure of unliganded HSV gD reveals a mechanism for receptor-mediated activation of virus entry EMBO J 2005 24 23 4144 4153 10.1038/sj.emboj.7600875 16292345 PMC1356314

[B35] Heldwein EE Lou H Bender FC Cohen GH Eisenberg RJ Harrison SC Crystal structure of glycoprotein B from herpes simplex virus 1 Science 2006 313 5784 217 220 10.1126/science.1126548 16840698

[B36] Lin E Spear PG Random linker-insertion mutagenesis to identify functional domains of herpes simplex virus type 1 glycoprotein B Proc Natl Acad Sci USA 2007 104 32 13140 13145 10.1073/pnas.0705926104 17666526 PMC1941792

[B37] Hannah BP Cairns TM Bender FC Whitbeck JC Lou H Eisenberg RJ Cohen GH Herpes simplex virus glycoprotein B associates with target membranes via its fusion loops J Virol 2009 83 13 6825 6836 10.1128/JVI.00301-09 19369321 PMC2698560

[B38] Galdiero S Vitiello M D'Isanto M Falanga A Cantisani M Browne H Pedone C Galdiero M The identification and characterization of fusogenic domains in herpes virus glycoprotein B molecules Chembiochem 2008 9 5 758 767 10.1002/cbic.200700457 18311743

[B39] Chowdary TK Cairns TM Atanasiu D Cohen GH Eisenberg RJ Heldwein EE Crystal structure of the conserved herpesvirus fusion regulator complex gH-gL Nat Struct Mol Biol 2010 17 7 882 888 10.1038/nsmb.1837 20601960 PMC2921994

[B40] Jackson JO Longnecker R Reevaluating herpes simplex virus hemifusion J Virol 2010 84 22 11814 11821 10.1128/JVI.01615-10 20844038 PMC2977880

[B41] Atanasiu D Saw WT Cohen GH Eisenberg RJ Cascade of events governing cell-cell fusion induced by herpes simplex virus glycoproteins gD, gH/gL, and gB J Virol 2010 84 23 12292 12299 10.1128/JVI.01700-10 20861251 PMC2976417

[B42] Trybala E Liljeqvist JA Svennerholm B Bergström T Herpes simplex virus types 1 and 2 differ in their interaction with heparan sulfate J Virol 2000 74 19 9106 9114 10.1128/JVI.74.19.9106-9114.2000 10982357 PMC102109

[B43] Gruenheid S Gatzke L Meadows H Tufaro F Herpes simplex virus infection and propagation in a mouse L cell mutant lacking heparan sulfate proteoglycans J Virol 1993 67 1 93 100 8380101 10.1128/jvi.67.1.93-100.1993 PMC237341

[B44] Nahmias AJ Kibrick S Inhibitory effect of heparin on herpes simplex virus J Bacteriol 1964 87 5 1060 1066 4289440 10.1128/jb.87.5.1060-1066.1964 PMC277146

[B45] WuDunn D Spear PG Initial interaction of herpes simplex virus with cells is binding to heparan sulfate J Virol 1989 63 1 52 58 2535752 10.1128/jvi.63.1.52-58.1989 PMC247656

[B46] Gerber SI Belval BJ Herold BC Differences in the role of glycoprotein C of HSV-1 and HSV-2 in viral binding may contribute to serotype differences in cell tropism Virology 1995 214 1 29 39 10.1006/viro.1995.9957 8525631

[B47] Cheshenko N Herold BC Glycoprotein B plays a predominant role in mediating herpes simplex virus type 2 attachment and is required for entry and cell-to-cell spread J Gen Virol 2002 83 Pt 9 2247 2255 12185280 10.1099/0022-1317-83-9-2247

[B48] Langeland N Holmsen H Lillehaug JR Haarr L Evidence that neomycin inhibits binding of herpes simplex virus type 1 to the cellular receptor J Virol 1987 61 11 3388 3393 2822948 10.1128/jvi.61.11.3388-3393.1987 PMC255933

[B49] Herold BC Visalli RJ Susmarski N Brandt CR Spear PG Glycoprotein C-independent binding of herpes simplex virus to cells requires cell surface heparan sulphate and glycoprotein B J Gen Virol 1994 75 Pt 6 1211 1222 8207388 10.1099/0022-1317-75-6-1211

[B50] Herold BC Spear PG Neomycin inhibits glycoprotein C (gC)-dependent binding of herpes simplex virus type 1 to cells and also inhibits postbinding events in entry Virology 1994 203 1 166 171 10.1006/viro.1994.1469 8030274

[B51] Herold BC Gerber SI Belval BJ Siston AM Shulman N Differences in the susceptibility of herpes simplex virus types 1 and 2 to modified heparin compounds suggest serotype differences in viral entry J Virol 1996 70 6 3461 3469 8648678 10.1128/jvi.70.6.3461-3469.1996 PMC190219

[B52] Laquerre S Argnani R Anderson DB Zucchini S Manservigi R Glorioso JC Heparan sulfate proteoglycan binding by herpes simplex virus type 1 glycoproteins B and C, which differ in their contributions to virus attachment, penetration, and cell-to-cell spread J Virol 1998 72 7 6119 6130 9621076 10.1128/jvi.72.7.6119-6130.1998 PMC110418

[B53] Tumova S Woods A Couchman JR Heparan sulfate proteoglycans on the cell surface: versatile coordinators of cellular functions Int J Biochem Cell Biol 2000 32 3 269 288 10.1016/S1357-2725(99)00116-8 10716625

[B54] Bacsa S Karasneh G Dosa S Liu J Valyi-Nagy T Shukla D Syndecan-1 and syndecan-2 play key roles in herpes simplex virus type-1 infection J Gen Virol 2011 92 Pt 4 733 743 21148276 10.1099/vir.0.027052-0 PMC3133699

[B55] Campadelli-Fiume G Arsenakis M Farabegoli F Roizman B Entry of herpes simplex virus 1 in BJ cells that constitutively express viral glycoprotein D is by endocytosis and results in degradation of the virus J Virol 1988 62 1 159 167 2824844 10.1128/jvi.62.1.159-167.1988 PMC250514

[B56] Johnson RM Spear PG Herpes simplex virus glycoprotein D mediates interference with herpes simplex virus infection J Virol 1989 63 2 819 827 2536105 10.1128/jvi.63.2.819-827.1989 PMC247755

[B57] Johnson DC Burke RL Gregory T Soluble forms of herpes simplex virus glycoprotein D bind to a limited number of cell surface receptors and inhibit virus entry into cells J Virol 1990 64 6 2569 2576 2159532 10.1128/jvi.64.6.2569-2576.1990 PMC249433

[B58] Brandimarti R Huang T Roizman B Campadelli-Fiume G Mapping of herpes simplex virus 1 genes with mutations which overcome host restrictions to infection Proc Natl Acad Sci USA 1994 91 12 5406 5410 10.1073/pnas.91.12.5406 8202498 PMC44004

[B59] Montgomery RI Warner MS Lum BJ Spear PG Herpes simplex virus-1 entry into cells mediated by a novel member of the TNF/NGF receptor family Cell 1996 87 3 427 436 10.1016/S0092-8674(00)81363-X 8898196

[B60] Croft M Co-stimulatory members of the TNFR family: keys to effective T-cell immunity? Nat Rev Immunol 2003 3 8 609 620 10.1038/nri1148 12974476

[B61] Krummenacher C Nicola AV Whitbeck JC Lou H Hou W Lambris JD Geraghty RJ Spear PG Cohen GH Eisenberg RJ Herpes simplex virus glycoprotein D can bind to poliovirus receptor-related protein 1 or herpesvirus entry mediator, two structurally unrelated mediators of virus entry J Virol 1998 72 9 7064 7074 9696799 10.1128/jvi.72.9.7064-7074.1998 PMC109927

[B62] Tiwari V Clement C Scanlan PM Kowlessur D Yue BY Shukla D A role for herpesvirus entry mediator as the receptor for herpes simplex virus 1 entry into primary human trabecular meshwork cells J Virol 2005 79 20 13173 13179 10.1128/JVI.79.20.13173-13179.2005 16189018 PMC1235852

[B63] Eberlé F Dubreuil P Mattei MG Devilard E Lopez M The human PRR2 gene, related to the human poliovirus receptor gene (PVR), is the true homolog of the murine MPH gene Gene 1995 159 2 267 272 10.1016/0378-1119(95)00180-E 7622062

[B64] Lopez M Eberlé F Mattei MG Gabert J Birg F Bardin F Maroc C Dubreuil P Complementary DNA characterization and chromosomal localization of a human gene related to the poliovirus receptor-encoding gene Gene 1995 155 2 261 265 10.1016/0378-1119(94)00842-G 7721102

[B65] Geraghty RJ Krummenacher C Cohen GH Eisenberg RJ Spear PG Entry of alphaherpesviruses mediated by poliovirus receptor-related protein 1 and poliovirus receptor Science 1998 280 5369 1618 1620 10.1126/science.280.5369.1618 9616127

[B66] Warner MS Geraghty RJ Martinez WM Montgomery RI Whitbeck JC Xu R Eisenberg RJ Cohen GH Spear PG A cell surface protein with herpesvirus entry activity (HveB) confers susceptibility to infection by mutants of herpes simplex virus type 1, herpes simplex virus type 2, and pseudorabies virus Virology 1998 246 1 179 189 10.1006/viro.1998.9218 9657005

[B67] Takai Y Nakanishi H Nectin and afadin: novel organizers of intercellular junctions J Cell Sci 2003 116 Pt 1 17 27 12456712 10.1242/jcs.00167

[B68] Lopez M Cocchi F Menotti L Avitabile E Dubreuil P Campadelli-Fiume G Nectin2alpha (PRR2alpha or HveB) and nectin2delta are low-efficiency mediators for entry of herpes simplex virus mutants carrying the Leu25Pro substitution in glycoprotein D J Virol 2000 74 3 1267 1274 10.1128/JVI.74.3.1267-1274.2000 10627537 PMC111461

[B69] Shukla D Liu J Blaiklock P Shworak NW Bai X Esko JD Cohen GH Eisenberg RJ Rosenberg RD Spear PG A novel role for 3-O-sulfated heparan sulfate in herpes simplex virus 1 entry Cell 1999 99 1 13 22 10.1016/S0092-8674(00)80058-6 10520990

[B70] O'Donnell CD Shukla D The Importance of Heparan Sulfate in Herpesvirus Infection Virol Sin 2008 23 6 383 393 10.1007/s12250-008-2992-1 19956628 PMC2778322

[B71] Tiwari V O'donnell C Copeland RJ Scarlett T Liu J Shukla D Soluble 3-O-sulfated heparan sulfate can trigger herpes simplex virus type 1 entry into resistant Chinese hamster ovary (CHO-K1) cells J Gen Virol 2007 88 Pt 4 1075 1079 17374750 10.1099/vir.0.82476-0

[B72] O'Donnell CD Kovacs M Akhtar J Valyi-Nagy T Shukla D Expanding the role of 3-O sulfated heparan sulfate in herpes simplex virus type-1 entry Virology 2010 397 2 389 398 10.1016/j.virol.2009.11.011 20004926 PMC3351100

[B73] Tiwari V Clement C Xu D Valyi-Nagy T Yue BY Liu J Shukla D Role for 3-O-sulfated heparan sulfate as the receptor for herpes simplex virus type 1 entry into primary human corneal fibroblasts J Virol 2006 80 18 8970 8980 10.1128/JVI.00296-06 16940509 PMC1563926

[B74] Banfield BW Leduc Y Esford L Schubert K Tufaro F Sequential isolation of proteoglycan synthesis mutants by using herpes simplex virus as a selective agent: evidence for a proteoglycan-independent virus entry pathway J Virol 1995 69 6 3290 3298 7745676 10.1128/jvi.69.6.3290-3298.1995 PMC189040

[B75] Bender FC Whitbeck JC Lou H Cohen GH Eisenberg RJ Herpes simplex virus glycoprotein B binds to cell surfaces independently of heparan sulfate and blocks virus entry J Virol 2005 79 18 11588 11597 10.1128/JVI.79.18.11588-11597.2005 16140736 PMC1212636

[B76] Satoh T Arii J Suenaga T Wang J Kogure A Uehori J Arase N Shiratori I Tanaka S Kawaguchi Y Spear PG Lanier LL Arase H PILRalpha is a herpes simplex virus-1 entry coreceptor that associates with glycoprotein B Cell 2008 132 6 935 944 10.1016/j.cell.2008.01.043 18358807 PMC2394663

[B77] Arii J Uema M Morimoto T Sagara H Akashi H Ono E Arase H Kawaguchi Y Entry of herpes simplex virus 1 and other alphaherpesviruses via the paired immunoglobulin-like type 2 receptor alpha J Virol 2009 83 9 4520 4527 10.1128/JVI.02601-08 19244335 PMC2668467

[B78] Wang J Fan Q Satoh T Arii J Lanier LL Spear PG Kawaguchi Y Arase H Binding of herpes simplex virus glycoprotein B (gB) to paired immunoglobulin-like type 2 receptor alpha depends on specific sialylated O-linked glycans on gB J Virol 2009 83 24 13042 13045 10.1128/JVI.00792-09 19812165 PMC2786847

[B79] Suenaga T Satoh T Somboonthum P Kawaguchi Y Mori Y Arase H Myelin-associated glycoprotein mediates membrane fusion and entry of neurotropic herpesviruses Proc Natl Acad Sci USA 2010 107 2 866 871 10.1073/pnas.0913351107 20080767 PMC2818916

[B80] Cao Z Gao Y Deng K Williams G Doherty P Walsh FS Receptors for myelin inhibitors: Structures and therapeutic opportunities Mol Cell Neurosci 2010 43 1 1 14 10.1016/j.mcn.2009.07.008 19619659

[B81] Vicente-Manzanares M Ma X Adelstein RS Horwitz AR Non-muscle myosin II takes centre stage in cell adhesion and migration Nat Rev Mol Cell Biol 2009 10 11 778 790 10.1038/nrm2786 19851336 PMC2834236

[B82] Arii J Goto H Suenaga T Oyama M Kozuka-Hata H Imai T Minowa A Akashi H Arase H Kawaoka Y Kawaguchi Y Non-muscle myosin IIA is a functional entry receptor for herpes simplex virus-1 Nature 2010 467 7317 859 862 10.1038/nature09420 20944748

[B83] Clement C Tiwari V Scanlan PM Valyi-Nagy T Yue BY Shukla D A novel role for phagocytosis-like uptake in herpes simplex virus entry J Cell Biol 2006 174 7 1009 1021 10.1083/jcb.200509155 17000878 PMC2064392

[B84] Oh MJ Akhtar J Desai P Shukla D A role for heparan sulfate in viral surfing Biochem Biophys Res Commun 2010 391 1 176 181 10.1016/j.bbrc.2009.11.027 19909728 PMC2812628

[B85] Bender FC Whitbeck JC Ponce de Leon M Lou H Eisenberg RJ Cohen GH Specific association of glycoprotein B with lipid rafts during herpes simplex virus entry J Virol 2003 77 17 9542 9552 10.1128/JVI.77.17.9542-9552.2003 12915568 PMC187402

[B86] Scanlan PM Tiwari V Bommireddy S Shukla D Cellular expression of gH confers resistance to herpes simplex virus type-1 entry Virology 2003 312 1 14 24 10.1016/S0042-6822(03)00176-4 12890617

[B87] Parry C Bell S Minson T Browne H Herpes simplex virus type 1 glycoprotein H binds to alphavbeta3 integrins J Gen Virol 2005 86 Pt 1 7 10 15604426 10.1099/vir.0.80567-0

[B88] Galdiero M Whiteley A Bruun B Bell S Minson T Browne H Site-directed and linker insertion mutagenesis of herpes simplex virus type 1 glycoprotein H J Virol 1997 71 3 2163 2170 9032350 10.1128/jvi.71.3.2163-2170.1997 PMC191323

[B89] Gianni T Cerretani A Dubois R Salvioli S Blystone SS Rey F Campadelli-Fiume G Herpes simplex virus glycoproteins H/L bind to cells independently of {alpha}V{beta}3 integrin and inhibit virus entry, and their constitutive expression restricts infection J Virol 2010 84 8 4013 4025 10.1128/JVI.02502-09 20147400 PMC2849490

[B90] Perez A Li QX Perez-Romero P Delassus G Lopez SR Sutter S McLaren N Fuller AO A new class of receptor for herpes simplex virus has heptad repeat motifs that are common to membrane fusion proteins J Virol 2005 79 12 7419 7430 10.1128/JVI.79.12.7419-7430.2005 15919898 PMC1143644

[B91] Cheshenko N Trepanier JB Segarra TJ Fuller AO Herold BC HSV usurps eukaryotic initiation factor 3 subunit M for viral protein translation: novel prevention target PLoS One 2010 5 7 e11829 10.1371/journal.pone.0011829 20676407 PMC2910742

[B92] Copeland R Balasubramaniam A Tiwari V Zhang F Bridges A Linhardt RJ Shukla D Liu J Using a 3-O-sulfated heparin octasaccharide to inhibit the entry of herpes simplex virus type 1 Biochemistry 2008 47 21 5774 5783 10.1021/bi800205t 18457417 PMC2504729

[B93] Tiwari V Liu J Valyi-Nagy T Shukla D Anti-heparan sulfate peptides that block herpes simplex virus infection in vivo J Biol Chem 2011 286 28 25406 25415 10.1074/jbc.M110.201103 21596749 PMC3137111

[B94] Wittels M Spear PG Penetration of cells by herpes simplex virus does not require a low pH-dependent endocytic pathway Virus Res 1991 18 2-3 271 290 10.1016/0168-1702(91)90024-P 1645908

[B95] Dollery SJ Delboy MG Nicola AV Low pH-induced conformational change in herpes simplex virus glycoprotein B J Virol 2010 84 8 3759 3766 10.1128/JVI.02573-09 20147407 PMC2849479

[B96] Tiwari V Oh MJ Kovacs M Shukla SY Valyi-Nagy T Shukla D Role for nectin-1 in herpes simplex virus 1 entry and spread in human retinal pigment epithelial cells FEBS J 2008 275 21 5272 5285 10.1111/j.1742-4658.2008.06655.x 18803666 PMC2758088

[B97] Nicola AV McEvoy AM Straus SE Roles for endocytosis and low pH in herpes simplex virus entry into HeLa and Chinese hamster ovary cells J Virol 2003 77 9 5324 5332 10.1128/JVI.77.9.5324-5332.2003 12692234 PMC153978

[B98] Stiles KM Milne RS Cohen GH Eisenberg RJ Krummenacher C The herpes simplex virus receptor nectin-1 is down-regulated after trans-interaction with glycoprotein D Virology 2008 373 1 98 111 10.1016/j.virol.2007.11.012 18076965 PMC2629994

[B99] Delboy MG Patterson JL Hollander AM Nicola AV Nectin-2-mediated entry of a syncytial strain of herpes simplex virus via pH-independent fusion with the plasma membrane of Chinese hamster ovary cells Virol J 2006 3 105 10.1186/1743-422X-3-105 17192179 PMC1779275

[B100] Nicola AV Straus SE Cellular and viral requirements for rapid endocytic entry of herpes simplex virus J Virol 2004 78 14 7508 7517 10.1128/JVI.78.14.7508-7517.2004 15220424 PMC434080

[B101] Gianni T Gatta V Campadelli-Fiume G {alpha}V{beta}3-integrin routes herpes simplex virus to an entry pathway dependent on cholesterol-rich lipid rafts and dynamin2 Proc Natl Acad Sci USA 2010 107 51 22260 22265 10.1073/pnas.1014923108 21135248 PMC3009828

[B102] Linehan MM Richman S Krummenacher C Eisenberg RJ Cohen GH Iwasaki A In vivo role of nectin-1 in entry of herpes simplex virus type 1 (HSV-1) and HSV-2 through the vaginal mucosa J Virol 2004 78 5 2530 2536 10.1128/JVI.78.5.2530-2536.2004 14963155 PMC369262

[B103] Schlitt M Lakeman AD Wilson ER To A Acoff RW Harsh GR Whitley RJ A rabbit model of focal herpes simplex encephalitis J Infect Dis 1986 153 4 732 735 10.1093/infdis/153.4.732 3005433

[B104] Bernstein DI Ireland J Bourne N Pathogenesis of acyclovir-resistant herpes simplex type 2 isolates in animal models of genital herpes: models for antiviral evaluations Antiviral Res 2000 47 3 159 169 10.1016/S0166-3542(00)00104-2 10974368

[B105] Burgos JS Ripoll-Gomez J Alfaro JM Sastre I Valdivieso F Zebrafish as a new model for herpes simplex virus type 1 infection Zebrafish 2008 5 4 323 333 10.1089/zeb.2008.0552 19133831

[B106] Sears AE McGwire BS Roizman B Infection of polarized MDCK cells with herpes simplex virus 1: two asymmetrically distributed cell receptors interact with different viral proteins Proc Natl Acad Sci USA 1991 88 12 5087 5091 10.1073/pnas.88.12.5087 1647025 PMC51816

[B107] Menotti L Lopez M Avitabile E Stefan A Cocchi F Adelaide J Lecocq E Dubreuil P Campadelli-Fiume G The murine homolog of human Nectin1delta serves as a species nonspecific mediator for entry of human and animal alpha herpesviruses in a pathway independent of a detectable binding to gD Proc Natl Acad Sci USA 2000 97 9 4867 4872 10.1073/pnas.97.9.4867 10781093 PMC18324

[B108] Shukla D Rowe CL Dong Y Racaniello VR Spear PG The murine homolog (Mph) of human herpesvirus entry protein B (HveB) mediates entry of pseudorabies virus but not herpes simplex virus types 1 and 2 J Virol 1999 73 5 4493 4497 10196354 10.1128/jvi.73.5.4493-4497.1999 PMC104343

[B109] Shworak NW Liu J Fritze LM Schwartz JJ Zhang L Logeart D Rosenberg RD Molecular cloning and expression of mouse and human cDNAs encoding heparan sulfate D-glucosaminyl 3-O-sulfotransferase J Biol Chem 1997 272 44 28008 28019 10.1074/jbc.272.44.28008 9346953

[B110] Cadwallader AB Yost HJ Combinatorial expression patterns of heparan sulfate sulfotransferases in zebrafish: I. The 3-O-sulfotransferase family Dev Dyn 2006 235 12 3423 3431 10.1002/dvdy.20991 17075882

[B111] Hubbard S Darmani NA Thrush GR Dey D Burnham L Thompson JM Jones K Tiwari V Zebrafish-encoded 3-O-sulfotransferase-3 isoform mediates herpes simplex virus type 1 entry and spread Zebrafish 2010 7 2 181 187 10.1089/zeb.2009.0621 20441522

[B112] Wagner EK Bloom DC Experimental investigation of herpes simplex virus latency Clin Microbiol Rev 1997 10 3 419 443 9227860 10.1128/cmr.10.3.419 PMC172928

[B113] Grest P Albicker P Hoelzle L Wild P Pospischil A Herpes simplex encephalitis in a domestic rabbit (Oryctolagus cuniculus) J Comp Pathol 2002 126 4 308 311 10.1053/jcpa.2002.0548 12056779

[B114] Weissenböck H Hainfellner JA Berger J Kasper I Budka H Naturally occurring herpes simplex encephalitis in a domestic rabbit (Oryctolagus cuniculus) Vet Pathol 1997 34 1 44 47 10.1177/030098589703400107 9150545

[B115] Liu T Khanna KM Carriere BN Hendricks RL Gamma interferon can prevent herpes simplex virus type 1 reactivation from latency in sensory neurons J Virol 2001 75 22 11178 11184 10.1128/JVI.75.22.11178-11184.2001 11602757 PMC114697

[B116] Knickelbein JE Khanna KM Yee MB Baty CJ Kinchington PR Hendricks RL Noncytotoxic lytic granule-mediated CD8+ T cell inhibition of HSV-1 reactivation from neuronal latency Science 2008 322 5899 268 271 10.1126/science.1164164 18845757 PMC2680315

[B117] Kopp SJ Banisadr G Glajch K Maurer UE Grünewald K Miller RJ Osten P Spear PG Infection of neurons and encephalitis after intracranial inoculation of herpes simplex virus requires the entry receptor nectin-1 Proc Natl Acad Sci USA 2009 106 42 17916 17920 10.1073/pnas.0908892106 19805039 PMC2764878

[B118] Renis HE Eidson EE Mathews J Gray JE Pathogenesis of herpes simplex virus types 1 and 2 in mice after various routes of inoculation Infect Immun 1976 14 2 571 578 184048 10.1128/iai.14.2.571-578.1976 PMC420920

[B119] Sekizawa T Openshaw H Encephalitis resulting from reactivation of latent herpes simplex virus in mice J Virol 1984 50 1 263 266 6321795 10.1128/jvi.50.1.263-266.1984 PMC255608

[B120] Gebhardt BM Halford WP Evidence that spontaneous reactivation of herpes virus does not occur in mice Virol J 2005 2 67 10.1186/1743-422X-2-67 16109179 PMC1208961

[B121] Dasgupta G Benmohamed L Of mice and not humans: How reliable are animal models for evaluation of herpes CD8(+)-T cell-epitopes-based immunotherapeutic vaccine candidates? Vaccine 2011 29 35 5824 5836 10.1016/j.vaccine.2011.06.083 21718746 PMC3159167

[B122] Chentoufi AA Dasgupta G Christensen ND Hu J Choudhury ZS Azeem A Jester JV Nesburn AB Wechsler SL BenMohamed L A novel HLA (HLA-A*0201) transgenic rabbit model for preclinical evaluation of human CD8+ T cell epitope-based vaccines against ocular herpes J Immunol 2010 184 5 2561 2571 10.4049/jimmunol.0902322 20124097 PMC3752373

[B123] Tiwari V Shukla SY Yue BY Shukla D Herpes simplex virus type 2 entry into cultured human corneal fibroblasts is mediated by herpesvirus entry mediator J Gen Virol 2007 88 Pt 8 2106 2110 17622611 10.1099/vir.0.82830-0

[B124] Akhtar J Tiwari V Oh MJ Kovacs M Jani A Kovacs SK Valyi-Nagy T Shukla D HVEM and nectin-1 are the major mediators of herpes simplex virus 1 (HSV-1) entry into human conjunctival epithelium Invest Ophthalmol Vis Sci 2008 49 9 4026 4035 10.1167/iovs.08-1807 18502984 PMC2569872

[B125] Shah A Farooq AV Tiwari V Kim MJ Shukla D HSV-1 infection of human corneal epithelial cells: receptor-mediated entry and trends of re-infection Mol Vis 2010 16 2476 2486 21139972 PMC2994737

[B126] Shukla SY Singh YK Shukla D Role of nectin-1, HVEM, and PILR-alpha in HSV-2 entry into human retinal pigment epithelial cells Invest Ophthalmol Vis Sci 2009 50 6 2878 2887 10.1167/iovs.08-2981 19234349 PMC3686556

[B127] Prandovszky E Horváth S Gellért L Kovács SK Janka Z Toldi J Shukla D Vályi-Nagy T Nectin-1 (HveC) is expressed at high levels in neural subtypes that regulate radial migration of cortical and cerebellar neurons of the developing human and murine brain J Neurovirol 2008 14 2 164 172 10.1080/13550280801898672 18444088

[B128] Guzman G Oh S Shukla D Engelhard HH Valyi-Nagy T Expression of entry receptor nectin-1 of herpes simplex virus 1 and/or herpes simplex virus 2 in normal and neoplastic human nervous system tissues Acta Virol 2006 50 1 59 66 16599187

[B129] Choudhary S Marquez M Alencastro F Spors F Zhao Y Tiwari V Herpes Simplex Virus Type-1 (HSV-1) Entry into Human Mesenchymal Stem Cells Is Heavily Dependent on Heparan Sulfate J Biomed Biotechnol 2011 2011 264350 21799659 10.1155/2011/264350 PMC3134178

